# Continuing education on child development for primary healthcare professionals: a prospective before-and-after study

**DOI:** 10.1590/1516-3180.2014.1324665

**Published:** 2014-05-20

**Authors:** Amira Consuêlo Melo Figueiras, Rosana Fiorini Puccini, Edina Mariko Koga Silva

**Affiliations:** I MD, PhD. Adjunct Professor, Discipline of Pediatrics, School of Medicine, Universidade Federal do Pará (UFPA), Belém, Pará, Brazil; II MD, PhD. Titular Professor, Department of Pediatrics, Escola Paulista de Medicina -Universidade Federal de São Paulo (EPM-Unifesp), São Paulo, Brazil; III MD, PhD. Physician in the Discipline of Emergency Medicine and Evidence-Based Medicine, Escola Paulista de Medicina - Universidade Federal de São Paulo (EPM-Unifesp), São Paulo, Brazil

**Keywords:** Child development, Primary health care, Health knowledge, attitudes, practice, Program evaluation, Education, continuing, Desenvolvimento infantil, Atenção primária à saúde, Conhecimentos, atitudes e prática em saúde, Avaliação de programas e projetos de saúde, Educação continuada

## Abstract

**CONTEXT AND OBJECTIVE:**

Children's developmental disorders are often identified late by healthcare professionals working in primary care. The aim of this study was to assess the impact of a continuing education program on child development, on the knowledge and practices of these professionals.

**DESIGN AND SETTING:**

Prospective single-cohort study (before-and-after study), conducted in the city of Belém, Pará , Brazil.

**METHODS:**

Two hundred and twenty-one professionals working in primary healthcare (82.2%) participated in a continuing education program on child development and were assessed before and after implementation of the program through tests on their knowledge of child development, consisting of 19 questions for physicians and 14 for nurses, and questionnaires on their professional practices.

**RESULTS:**

One to three years after the program, the mean number of correct answers in the tests had increased from 11.5 to 14.3 among physicians in the Healthy Family Program (Programa Família Saudável, PFS); 13.0 to 14.3 among physicians in Municipal Health Units (Unidades Municipais de Saúde, UMS); 8.3 to 10.0 among PFS nurses; and 7.8 to 9.4 among UMS nurses. In interviews with mothers attended by these professionals before the program, only 21.7% reported that they were asked about their children's development, 24.7% reported that the professional asked about or observed their children's development and 11.1% received advice on how to stimulate them. After the program, these percentages increased to 34.5%, 54.2% and 30.3%, respectively.

**CONCLUSIONS:**

Professionals who participated in the program showed improved performance regarding child development knowledge and practices.

## Abstract (pt)

**CONTEXTO E OBJETIVO:**

Alterações do desenvolvimento em crianças frequentemente têm sido tardiamente identificadas por profissionais de saúde que atuam na atenção básica. O objetivo deste estudo foi avaliar o impacto de um programa de educação permanente sobre desenvolvimento infantil nos conhecimentos e práticas desses profissionais.

**TIPO DE ESTUDO E LOCAL:**

Estudo de coorte única prospectivo (antes-e-depois), realizado no município de Belém, Pará, Brasil.

**MÉTODOS:**

221 (82,2%) profissionais da rede básica de saúde que participaram do programa de educação permanente sobre desenvolvimento infantil foram avaliados antes e após a implantação do programa, através de testes sobre seus conhecimentos em desenvolvimento infantil com 19 questões para médicos e 14 para enfermeiros, e questionários sobre sua prática profissional.

**RESULTADOS:**

Um a três anos após o programa, a média de perguntas certas dos testes aumentou de 11,5 para 14,3 entre os médicos do Programa da Família Saudável (PFS); 13,0 para 14,3 entre os médicos de Unidades Municipais de Saúde (UMS); 8,3 para 10,0 entre os enfermeiros de PFS e 7,8 para 9,4 entre os enfermeiros de UMS. Nas entrevistas com mães atendidas por esses profissionais, verificou-se que, antes do programa, apenas 21,7% informaram que foram indagadas sobre o desenvolvimento dos seus filhos, 24,7% relataram que o profissional perguntou ou observou o desenvolvimento da sua criança e 11,1% receberam orientação sobre como estimulá-las; após o programa, esses percentuais aumentaram para 34,5%, 54,2% e 30,3%, respectivamente.

**CONCLUSÃO:**

Profissionais que participaram do programa apresentaram melhor desempenho quanto aos conhecimentos e práticas sobre desenvolvimento infantil.

## INTRODUCTION

Child development is an extremely complex process that has a variety of different approaches and perspectives. Its conceptualization varies according to the theoretical framework that is to be adopted and the aspects that are to be addressed. Child development exhibits universal characteristics that are present in all children, regardless of culture or experiences, individual characteristics that are related to genetic inheritance, and characteristics that are related to family background and social conditions. Understanding these three aspects - universal patterns, individual characteristics and the influence of family and social context - is necessary in order to clearly understand each child's development and the approaches and interventions to be adopted in the face of developmental disorders. 

The first years of life, in particular, are the most important for full development, and the experiences accumulated during this period have an influence throughout life. Nerve tissue presents its highest growth and maturation during this period and is therefore more susceptible to injury. Because of children's high degree of plasticity, it is also at this stage that they respond best to treatments and environmental stimuli.^1^ ^-^ ^5^ However, to receive these interventions, it is necessary to identify the disorder and direct the child to specialized services at the earliest possible opportunity. Therefore, the task of professionals working in primary healthcare is to monitor the development of all children and identify those with specific needs in order to ensure timely referral for intervention.

In Brazil, actions aimed at monitoring child development institutionally have been implemented and improved over recent years.^6^ Nonetheless, even in the southern and southeastern regions - areas with higher socioeconomic status and better access to health services - child development monitoring has not been effective within primary health care.^7^ Studies conducted in Belém, state of Pará (PA), in the northern region, showed that even children with moderate and severe disabilities were referred late for specialized treatment and that primary care professionals did not have satisfactory development of their monitoring knowledge and practices.^8^ Based on these studies, the Municipal Health Department of Belém developed a project that was aimed at primary care professionals, i.e. physicians and nurses, in order to provide training in child development and expansion of the network of services available for children with preestablished developmental disorders. Other institutions - the Federal University of Pará (Universidade Federal do Pará, UFPA, the State Health Department of Pará, Pará Society of Pediatrics (Sociedade Paraense de Pediatria, SOPAPE), the Pan-American Health Organization and the United Nations Children's Fund (UNICEF) - were involved in this process, providing funding and technical support, and putting the project into operation. This effort later became the Child Development Monitoring Program (Programa de Vigilância do Desenvolvimento Infantil).^9^ ^-^ ^11^

There is little literature regarding training for health professionals for monitoring child development, and this is therefore an open research field. Studies that examine the effectiveness of different types of interventions in improving health professionals' performance have mostly shown that the results are related to the teaching methodologies used and that interactive activities should be prioritized, such as workshops in small groups and individualized training sessions.^12^ ^,^ ^13^ It has also been found that refresher courses, meetings, conferences, seminars, lectures and symposia are ineffective for improving physicians' performance. 

## OBJECTIVE

The present study aimed to describe the process involved in training Belém's primary healthcare professionals in child development and to analyze the knowledge and practices of the professionals who participated in the program, one to three years after its implementation. 

## METHODS

A prospective single-cohort study (before-and-after study) was conducted in the city of Belém, state of Pará, located in northern Brazil. As the capital of the second largest state in Brazil, Belém had 29 primary healthcare units during the study period, known as the Municipal Health Units (Unidades Municipal de Saúde, UMS), two teaching health centers linked to the Federal and State Universities of Pará and 29 Healthy Family Centers, where 86 Healthy Family Program (Programa Família Saudável, PFS) teams and 22 community agents worked. Specialized services for children with disabilities included a state referral unit, two university referral units (one in partnership with the Municipal Health Department), one municipal unit, two philanthropic units and two privately-owned units that had service agreements with the National Health System (Sistema Único de Saúde, SUS). Of the 29 UMS units, 18 participated in this study, and the remaining 11 were administered by the state at the time when the study began.^10^

In 2001, the Municipal Health Department of Belém incorporated the Child Development Monitoring in Primary Healthcare Project into its programming.^11^ This professional training program was aimed at physicians and nurses of the UMS and PFS units and included an on-site course of 20 hours. It made use of the adapted clinical manual for Integrated Management of Childhood Illness (IMCI) (or AIDPI: Atenção Integrada às Doenças Prevalentes na Infância, in Portuguese), which was later published by the Pan-American Health Organization (PAHO) under the title "Monitoring Child Development in the IMCI Context".^14^ ^,^ ^15^ This handbook addresses the importance of monitoring child development, the risk factors for developmental delay, the acquisition of psychomotor development milestones and guidelines on stimulation and procedures when detecting any delay or disorder in the development of children up to two years of age. The course program included lectures, discussions on topics relating to normal development and disorders in child development, readings, videos and practical assessments with children using the methodology presented in the handbook*. *Later on, the professionals also attended three meetings with the program coordinators, where they discussed the cases that had been referred to them and issues relating to the referred cases, with an emphasis on cerebral palsy, autism and the development of preterm newborns. 

One to three years after completion of the program, an assessment was conducted to investigate the knowledge acquired and changes to practices among these healthcare professionals with regard to child development. A consent form was drafted for distribution among the professionals and mothers who were assessed, which explained the research aims for them. The research stages and materials were reviewed and approved by the Research Ethics Committee of Unifesp.

### Assessment tools

The professionals' knowledge was assessed by applying the Child Development Test at three different time-points: before training (Pre-Test), immediately after training (Immediate Post-Test) and one to three years after the training (One-year Post-Test). Their practices were assessed by applying a questionnaire on practices relating to child development monitoring (Practice-Quest), which was applied before the training (Pre-Practice-Quest) and one to three years after the training (One-year Post-Practice-Quest) and also through interviews with mothers, which were conducted immediately after the child's consultation and asked about the professional's assessment and guidance regarding the child's development. These interviews with the mothers were conducted before the professionals took part in the program (Pre-Int-mothers) and 1-3 years after completion (One-year Post-Int-mothers). 

The Child Development Test comprised 19 objective questions relating to child development, including motor, language, personal-social and cognitive skills in the first years of life. These questions identified risk factors for developmental problems and diseases that interfered with the child's normal development. The test was developed by pediatricians who worked in the care program for children with developmental risk and/or delay at the Paulista School of Medicine, Federal University of São Paulo (Universidade Federal de São Paulo, Unifesp) and was applied to the 25 pediatric residents in this institution, comprising 13 R1 (first-year residents) and 12 R2 (second-year residents), after they had completed an internship in this program. The questions that produced score percentages below 80% were then revised and reformulated. Three models were developed with similar questions and difficulty levels for the Pre-Test, Immediate Post-Test and One-year Post-Test. Questions on diagnosing diseases relating to development were not included for nurses. Thus, only 14 questions were taken into consideration for these participants.

The Practice-Quest consisted of 12 questions relating to the professional's childcare practices. In addition to seeking information on the interviewees' identification number, profession, education, prior specialization, employment contracts and use of child development assessment tools, the questionnaire also included three questions relating to the following professional childcare practices that were analyzed in the present study: 1) Do you usually ask mothers what they think about their children's development? 2) Do you conduct routine assessments on the development of children attending this service? and 3) Do you usually advise mothers on how to stimulate their children's development? 

The interviews with the mothers were conducted within the health services immediately after the appointment with the physician or nurse, and these professionals did not know about the content of the interviews that were conducted. The interviews contained 11 questions and were carried out at two different time-points. They sought information on the identification number, child's date of birth, enrollment data, consultation type (scheduled or not) and the following three questions relating to professional practice: 1) whether the professional asked the mother what she thought about her child's development; 2) whether the professional observed the child's behavior; and 3) whether the professional guided the mother on how to stimulate her child's development. The purpose of these questions was to use information from the mother to assess the professional's attitude regarding the monitoring of child development during the consultation. The interviews were conducted by a team consisting of six people, including a physician, two nurses, two physical therapy students and a nursing technician. The team had previously been provided with guidance and training so that they maintained uniformity in the data completion. The interviews with the mothers were conducted on a convenience sample, regardless of whether this was a scheduled childcare appointment. 

A database was created using Epi Info version 6.0 4b, for storage and analysis of the information gathered.^16^ Chi-square or Fisher's tests were used to compare categorical variables between the two groups. Both were calculated using the Epitable program of Epi Info 6.01.^17^ The analysis of variance method was used to calculate and compare the means in the Epitable program of Epi Info 6.01). In all statistical tests, a significance level of 5% (x = 0.05) was used. Bold type was used to indicate when the calculated P value (minimum significance level) allowed the null hypothesis to be rejected (P < 0.05).

## RESULTS

During the implementation of the program, 269 professionals were working in primary care units in the municipality of Belém. In the initial stage of the research, an assessment was made of the knowledge and practices at consultations among 221 professionals (82.2% of the total number): 30 UMS physicians (66.6%), 59 PSF physicians (68.6%), 49 UMS nurses (94.2%) and 83 PSF nurses (96.5%). In the final stage of the study (one to three years later), some of these professionals were no longer working in the municipality's primary care network. Thus, 136 professionals participated in the knowledge assessment (in one of the continuing education meetings) and 156 participated in the practice assessment (in the health units). A total of 442 mothers participated in the initial stage of the study (two mothers per professional). In the final stage, 264 of the 312 referred mothers (84.6%) agreed to participate in the study (Table 1). 

**Table 1:** Tools applied according to professional category, Belém, 2004

Professional	PFS physicians		UMS physicians		PFS nurses		UMS nurses		Total	
	n	%	n	%	n	%	n	%	n	%
Pre-Test	59	100.0	30	100.0	83	100.0	49	100.0	221	100.0
Immediate Post-Test	59	100.0	30	100.0	83	100.0	49	100.0	221	100.0
One-year Post-Test	28	48.0	21	70.0	62	75.0	25	51.0	136	61.0
Pre-Practice-Quest	59	100.0	30	100.0	83	100.0	49	100.0	221	100.0
One-year Post-Practice-Quest	35	59.0	24	80.0	70	84.0	27	55.0	156	71.0
Pre-Int-mothers	118	100.0	60	100.0	166	100.0	98	100.0	442	100.0
One-year Post-Int-mothers	58	49.0	44	73.0	112	67.0	50	51.0	264	60.0
Total	416		239		659		347		1661	

UMS = Unidades Municipais de Saúde (Municipal Health Units)

PFS = Programa da Família Saudável (Healthy Family Program)

The results relating to the professionals' knowledge about child development are shown in Table 2 as the means of the correct answers in the Pre-Test, Immediate Post-Test and One-year Post-Test, organized according to job category and work location. All 221 professionals answered the Pre-Test and Immediate Post-Test. The One-year Post-Test was completed by 136 professionals who remained in the program for over a year and who attended the continuing education meeting at which the One-year Post-Test was applied. There were statistically significant increases in the mean numbers of correct responses between the Pre-Test and Immediate Post-Test and between the Pre-Test and the One-year Post-Test in all professional categories. Comparison between the Immediate Post-Test and the One-year Post-Test did not show any statistically significant difference.

**Table 2:** Means for correct answers in pre-test, immediate post-test and one-year post-test, according to professional category. Belém, 2004

Professional	Pre-Test		Immediate Post-Test		One-year Post-Test		Pre-Test versus Immediate Post-Test	Pre-Test versus One-year Post-Test	Immediate Post-Test versus One-year Post-Test
	Mean	Variance	Mean	Variance	Mean	Variance			
PFS physicians	11.5	5.6	14.7	5	13.7	5	P < 0.001	P < 0.001	P = 0.05
UMS physicians	13	3.8	14.9	5.8	14.3	4	P < 0.001	P < 0.001	P = 0.35
PFS nurses	8.3	4.7	10.1	3.9	10	3.9	P < 0.001	P < 0.001	P = 0.76
UMS nurses	7.8	4.9	10.1	2.9	9.4	2.9	P < 0.001	P = 0.002	P = 0.09

**Figure 1:**
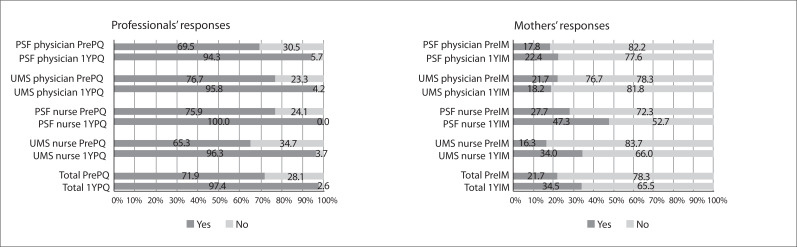
Answers to the question "Did the professional ask the mother what she thought about her child's development?"

**Figure 2:**
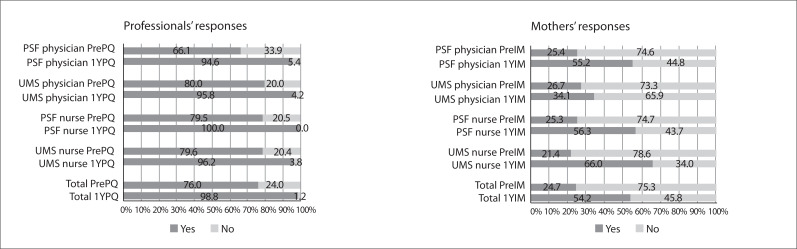
Answers to the question "Did the professional routinely assess the child's development?"

**Figure 3:**
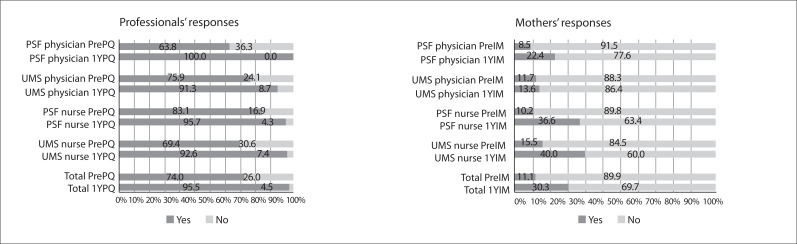
Answers to the question "Did the professional guide the mother about how to stimulate her child's development?"

Figures 1, 2 and 3 show the results for the professionals' and accompanying mothers' responses to the three professional practices questions and the differences between the responses of the professionals and the mothers for each question. When asked "Do you ever ask the mothers what they think about their children's development?", 159 of the professionals (71.9%) answered "yes" in the Pre-Practice-Quest, compared with 152 (97.4%) in the One-year Post-Practice-Quest. With the exception of the physicians in the UMS units (30 professionals), there was a statistically significant difference between the responses at the first and second time-points. However, among the Pre-Int-mothers*,* only 21.7% of the mothers reported that the professional asked their opinion on their child's development, while among the One-year Post-Int-mothers*,* 34.5% of the mothers answered "yes" to this question, thus showing a statistically significant difference. Analysis according to professional category did not reveal any statistically significant difference between physicians at the PFS units and UMS units (Figure 1). In interviews with mothers whose children attended scheduled consultations (261), the percentages were slightly higher, with no statistically significant difference: 22.6% among the Pre-Int-mothers and 41.1% among the One-year Post-Int-mothers.

In response to the question asking the professional whether he/she conducted routine assessment of children's development during consultations (Figure 2), 168 (76%) answered "yes" in the Pre-Practice-Quest*,* and 151 (98.8%) in the One-year Post-Practice-Quest. With the exception of UMS physicians and nurses, there was also a statistically significant difference between the professionals' responses to the first and second Practice-Quest. According to information from the mothers, among the Pre-Int-mothers, 24.7% of the professionals asked about or observed the child's behavior, and among the One-year Post-Int-mothers, this proportion was 54.2%. This difference was statistically significant, with the only exception being the UMS physicians. In scheduled consultations, this percentage increased from 29.0% to 65.2%.

Regarding the question "Do you usually give mothers guidelines on how to stimulate their children's development?", 74.0% answered "yes" in the Pre-Practice-Quest, and 95.5% answered "yes" in the One-year Post-Practice Quest. This difference was statistically significant for all professional categories except for the UMS physicians. Among the Pre-Int-mothers, only 11.1% of the mothers interviewed stated that they had received some form of professional guidance on how to stimulate their child's development. Among the One-year Post-Int-mothers, this percentage increased to 30.3%, which was a statistically significant increase, except among the UMS physicians (Figure 3). In interviews with the mothers of children with scheduled appointments, the percentage increased from 15.3% among the Pre-Int-mothers to 34.8% among the One-year Post-Int-mothers.

## DISCUSSION

The aim of this study was to contribute towards the process of reorienting healthcare actions that are focused on monitoring child development and also to obtain knowledge of teaching-learning methodologies that are geared towards healthcare professionals.

The assessment on the professionals prior to training, which was achieved by applying the pre-test, showed that they had inadequate levels of knowledge regarding child development, in all professional categories but most markedly among the nurses. Della Barba applied the same tool as used in this pre-test to 65 first and second-year pediatric residents in São Paulo and obtained higher mean scores (13.9; 73.2%) than those achieved by the physicians in the present study, which was conducted in Belém.^18^ This outcome may have arisen because the São Paulo residents were pursuing specialization in pediatrics and might have had more taught content relating to development of practice and academic activities within medical residency, whereas not all of the Belém physicians had a residency/specialization in pediatrics. Other studies have also shown gaps in physicians' knowledge of child development.^19^ ^,^ ^20^ In assessing the training of PSF nurses in Fortaleza, Ceará, for early detection of psychomotor disorders in breastfed infants, Nobrega et al. also found that most nurses considered themselves to be ill-prepared for this function.^21^ The nurses' difficulty was also observed by other authors.^22^ ^,^ ^23^

In the Immediate Post-Test*, *conducted at the end of the Child Development Monitoring Course, a statistically significant improvement in knowledge could be seen. The mean percentage of correct answers exceeded 70%, for questions in all professional categories, thus showing that there had been good assimilation of the content addressed in the course. In the post-test performed one to three years after the course (One-year Post-Test), it could be seen that the knowledge acquired had been retained in relation to the Immediate Post-Test. The difference was not statistically significant, and a mean percentage of correct answers below 70% was only found among the UMS nurses. These data show that the knowledge acquired was retained for a period of one to three years. Reichert used the same assessment tool as in the present study as well as the "Monitoring Child Development in the IMCI Context" handbook, in training for 45 nurses in PFS teams in the city of João Pessoa, Northeastern Brazil.^15^ ^,^ ^24^ The author observed a statistically significant improvement in child development knowledge four months after the program, with the percentage of correct answers increasing from 56.2% to 65.4%.

In Turkey, Ertem et al. assessed a development training process for physicians and nurses in primary care and found an improvement in the knowledge and practices of these professionals in an immediate assessment and in one conducted a year after the training.^25^ Other studies have also demonstrated the effectiveness of child development training processes that are aimed at medical students and primary healthcare professionals, in institutional programs that ensure not only health promotion and disability prevention but also care for children who have been identified as presenting a problem in their development.^26^ ^,^ ^27^

Parents' opinions regarding their children's development have been recognized by many researchers as having high sensitivity and specificity for detecting possible disorders.^28^ ^-^ ^31^ However, primary care professionals do not always fully appreciate parental complaints regarding possible problems in their children's development. In the present study, most of the professionals stated that they asked mothers what they thought about their children's development. However, less than half of the mothers gave affirmative answers to this question. PFS nurses were the ones who most frequently incorporated this type of approach among the mothers after the program had been implemented, possibly because of their increased awareness of the importance of sharing with the parents the perception of their child's development. This relationship seems to be more emphasized in nursing courses, and it also needs to be adopted by physicians. Other research studies also seem to corroborate these findings.^23^ ^,^ ^24^

When the professionals were asked whether they routinely assessed the child's development during the consultation, most of them also answered "yes", while less than half of the mothers perceived that their children's development was being assessed. This percentage increased to 54.2% after the program and to 65% when considering only the children who attended scheduled appointments. The changes were more significant among the UMS nurses. It is possible that the mothers did not notice that their children's development was being assessed because of a lack of proper communication. In relation to the same question, Ribeiro et al. found that 51.9% of the mothers attended by UMS physicians answered "yes",^32^ and Reichert recorded that 48% gave affirmative answers before and 79.1% after the program, in a study on consultations provided by PFS nurses.^24^

In a study conducted by Halfon et al. among mothers attended by pediatricians in the United States, it was found that 57% of the children aged between 10 and 35 months old received developmental assessment, and that 45% of the parents remembered that the pediatrician told them that he/she was assessing their child's development, while 39% stated that their child was assessed using some type of procedure that was included in the developmental assessment.^33^ It is noteworthy that in the United States, developmental assessment is mandated by law.^34^

With regard to guidance for mothers on their children's development, most of the professionals said that this was performed. For this same question, however, the mothers showed a much lower percentage of affirmative responses, even when only the scheduled consultations were taken into consideration. The UMS nurses presented the largest change in their attitudes regarding this item after training. In investigating this same question, Ribeiro et al. and Reichert also found that lower percentages of the mothers reported having received guidance on their children's development during their appointments.^24^ ^,^ ^32^ Thus, it is possible to conclude that the health professionals' awareness of the importance of providing mothers with guidance on their children's development increased after the training process and that, in general, nurses showed better performance of this activity than did physicians. 

These findings allow us to make two observations. Firstly, regarding the physician's role in monitoring child development, irrespective of whether this is a family physician or a pediatrician working in a primary healthcare unit, the findings from the present study suggest that better knowledge does not always result in better practices. Secondly, it can be seen, from this and other studies conducted in different regions of the country, that there is insufficient knowledge about child development. It is also worth noting that various work conditions and processes play a role in these healthcare units in Belém, namely the higher demands placed on UMS physicians by children with clinical complications and the salary differences that are dependent upon whether the professional works for the UMS or for the PFS.

Taking into account the profound changes in families, society and healthcare that have taken place over recent decades, children and adolescents now present new demands and health needs. In this context, constant updates to the search for appropriate and timely responses are thus essential and should include appropriate training and professional qualification, and organization of health services such that they promote integration, connection and continuity of care.

One limitation in this study that may have had some influence on the final result from evaluating the professionals one to three years afterwards was that was that some of the professionals in the study group were no longer present. To deal with this, one suggestion would be to keep track of a larger number of professionals, so that only the ones still present after working for at least one year in primary health care would be assessed.

## CONCLUSIONS

One positive conclusion that can be drawn from the present study, and which is supported by the literature, is that well-designed training programs and continuing education, with appropriate methodologies, can improve health professionals' knowledge in this field. It could be concluded from the present study that the professional training process, involving not only theoretical training but also practice and follow-up meetings to discuss cases, increased the knowledge among professionals in all categories, and that this knowledge was retained over time. It is essential to maintain this process of continuing education, so that this knowledge can further increase and transform into professional practice.
